# The Role of Sirtuin 6 in the Deacetylation of Histone Proteins as a Factor in the Progression of Neoplastic Disease

**DOI:** 10.3390/ijms25010497

**Published:** 2023-12-29

**Authors:** Marzena Baran, Paulina Miziak, Andrzej Stepulak, Marek Cybulski

**Affiliations:** Department of Biochemistry and Molecular Biology, Medical University of Lublin, 1 Chodzki Street, 20-093 Lublin, Poland; marzenabaran@umlub.pl (M.B.); paulina.miziak@gmail.com (P.M.); marekcybulski@umlub.pl (M.C.)

**Keywords:** sirtuins, histone, deacetylation, SIRT6 inhibitors, SIRT6 activators

## Abstract

SIRT6 is a nicotinamide adenine dinucleotide (NAD^+^)-dependent deacetylase, predominantly located in the nucleus, that is involved in the processes of histone modification, DNA repair, cell cycle regulation, and apoptosis. Disturbances in SIRT6 expression levels have been observed in the development and progression of various types of cancer. Therefore, it is important to better understand the role of SIRT6 in biochemical pathways and assign it specific biological functions. This review aims to summarize the role of SIRT6 in carcinogenesis and tumor development. A better understanding of the factors influencing SIRT6 expression and its biological role in carcinogenesis may help to develop novel anti-cancer therapeutic strategies. Moreover, we discuss the anti-cancer effects and mechanism of action of small molecule SIRT6 modulators (both activators and inhibitors) in different types of cancer.

## 1. Introduction

Numerous post-translational modifications alter the interaction between DNA, histone proteins, transcription factors (TFs), and chromatin. These modifications include acetylation, methylation, phosphorylation, ubiquitination, sumoylation, and ADP-ribosylation [1]. Acetylation and deacetylation are important mechanisms responsible for the regulation of gene transcription through the modulation of interactions of DNA with histone and non-histone proteins [2,3]. They are catalyzed by histone acetyltransferases (HATs) and histone deacetylases (HDACs), respectively. HATs catalyze the transfer of acetyl groups from acetyl-coenzyme A (Acetyl-CoA) to the ε-amino group of lysine residues in target proteins. Deacetylation of acetylated proteins is carried out by HDACs, which catalyze the hydrolytic removal of acetyl groups from proteins’ lysine residues [4]. Histone acetylation and deacetylation have a significant impact on numerous processes, including gene transcription, cell division, differentiation, DNA repair, and autophagy [4,5]. Therefore, the balance between HAT/HDAC activity plays an important role in regulating gene expression and cell signaling [4].

Sirtuins (SIRTs) are a family of enzymes belonging to class III of histone deacetylases, which includes seven members (SIRT1-7) [6]. All of them have histone deacetylation activity in the presence of nicotine adenine dinucleotide (NAD^+^) that is converted to nicotinamide (NAM) during this reaction. NAM can be converted back to NAD^+^ or methylated by nicotinamide N-methyltransferase (NNMT), which removes NAM from the NAD salvage pathway. Therefore, NNMT activity affects the availability of NAD^+^ for reactions carried out by SIRTs that influence sirtuin-mediated body homeostasis [7]. SIRTs are involved in numerous processes occurring in the cell. They participate in the process of maintaining homeostasis, genome stability, responses to stress, and aging [8]. Therefore, disruption of the activity of SIRTs and factors regulating their function, such as NAD^+^ and NNMT, contributes to the development of pathological conditions, inflammation, the deregulation of metabolic processes, neuronal diseases and disorders of cell proliferation, and apoptosis, leading to the development of cancer [6,8,9]. Therefore, SIRT modulators may have potential applications in the treatment of diseases such as Alzheimer’s disease (AD), Parkinson’s disease (PD), Huntington’s disease (HD), breast cancer (BC), head and neck squamous cell carcinoma (HNSCC), bladder cancer (BC), prostate cancer (PC), and renal cell carcinoma (RCC) [6,7,8].

This review presents the current knowledge on SIRT6, a member of the sirtuin family, and it aims to summarize the role of SIRT6 in carcinogenesis and tumor development. A better understanding of factors influencing SIRT6 expression and its biological role in carcinogenesis may help to develop novel anti-cancer therapeutic strategies. Moreover, we discuss the anti-cancer effects and mechanism of action of small molecule SIRT6 modulators (both activators and inhibitors) in different types of cancer. This review concentrates solely on SIRT6, highlighting the importance of this lesser-known sirtuin. Investigating the regulation of SIRT6 function through low-molecular-weight compounds that either activate or inhibit its activity presents a promising avenue for therapeutic intervention.

### Overview of the Sirtuin Deacetylases

**Figure 1:**
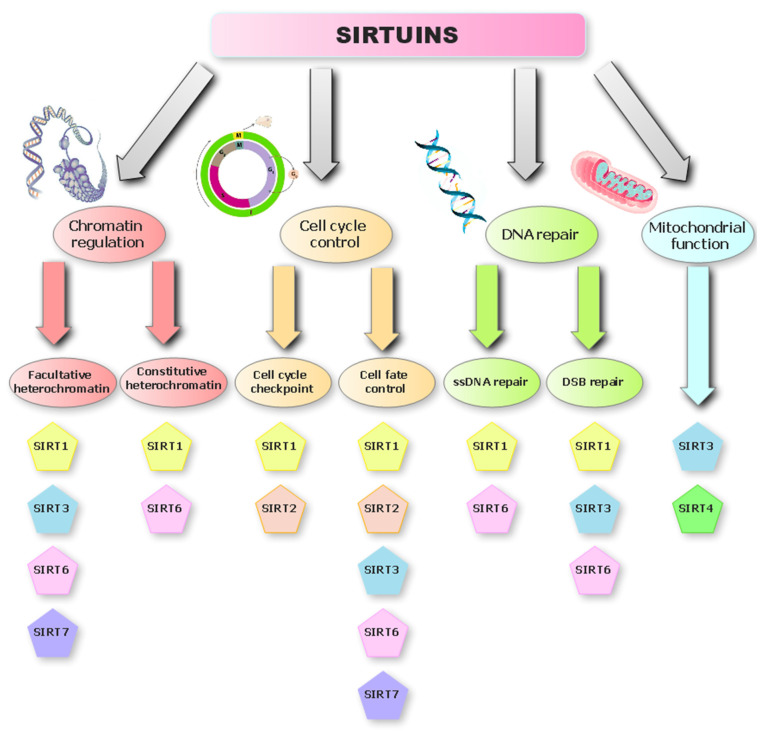
The role of sirtuins in the mechanisms of genomic stability.

There are four classes of histone deacetylases (HDACs), divided according to the substrate of the reaction they catalyze. Classes I, II, and IV use zinc ions as a cofactor in the reaction, and class III requires NAD^+^ for catalysis [10]. SIRTs belong to a family of highly conserved proteins that play an important role in the profiling of cell apoptosis, cell viability, tumorigenesis, and metastasis [11]. Currently, seven SIRT enzymes are known in mammals, which have been divided into four classes. All of them are homologues of yeast Sir2 and show nicotinamide adenine dinucleotide (NAD^+^)-dependent deacetylase and mono-ADP-ribosyl transferase activities. All SIRTs have a conserved domain that binds NAD^+^; however, they differ in their carboxyl and amino-terminal domains, which affects their catalytic activity and cell localization [12]. SIRTs remove the acyl group attached to the amino group of lysine residues present in acylated proteins, and for this process, they use one NAD^+^ molecule for each molecule of the removed acyl chain. As a result, three products are released: acylated ADP-ribose, deacylated protein, and nicotinamide [13]. NAD^+^ is a very important coenzyme in numerous metabolic transformations, which makes SIRTs essential regulators of the biochemical pathways responsible for cell division, aging, and the neoplastic process (Figure 1) [8,14]. SIRTs occur in various cellular compartments; SIRT1, 2, 6, 7 are nuclear proteins, but SIRT2 can move to the cytoplasm, and SIRT3, 4, 5 are located only in the mitochondrion [15]. This is probably related to their space structures, as they differ in the sequence of amino acids and the length of the chains in the N- and C-terminal domains. This affects their enzymatic activity in binding substrates [6]. All sirtuins have a catalytic domain that binds to (NAD^+^), which enables them to perform NAD^+^-dependent lysine deacetylation. However, it has been shown that some of them can remove acyl, malonyl, glutaryl, succinyl, and long-chain fatty residues [16]. SIRT1, 2, 3 were found to have greater deacetylation activity than SIRT4, 5, 6 [8].

## 2. SIRT6 Structure

**Figure 2:**
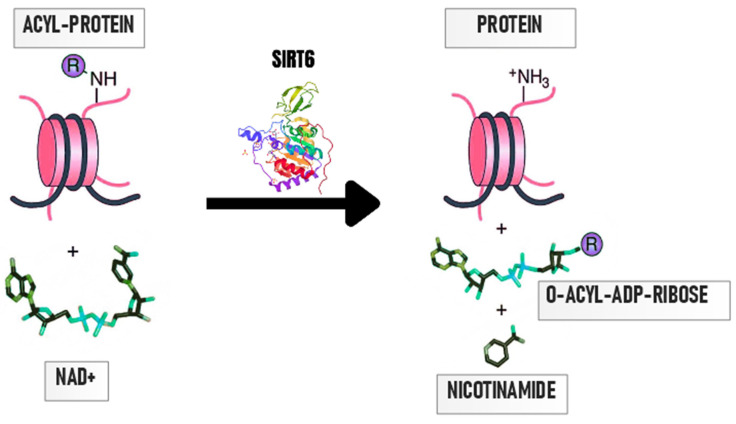
SIRT6 is an NAD^+^-dependent histone deacetylase. As a result of the attachment of NAD^+^ to SIRT6, the acyl group is removed from the protein. The reaction produces nicotinamide, O-acyl-ADPribose, and deacylated proteins [13].

One of the lesser-studied SIRTs is sirtuin 6 (SIRT6), an enzymatic protein belonging to class I sirtuins, located in the cell nucleus [12,17]. SIRT6 consists of 355 amino acids [10], of which 275 form a catalytic core that contains two spherical domains of eight α-helices and nine β-strands: the large Rossmann domain (residues 25–128 and 191–266) and a small zinc-binding domain-containing zinc with four cysteine residues, Cys141, Cys144, Cys166, and Cys177 [18,19], which form a specific structure responsible for the binding of the NAD^+^ coenzyme [13]. The large Rossmann domain is made up of six β-sheets (β1, β2, β3, β7, β8, and β9), which are located between six α helices (α1, α4, α5, α6, α7, and α8). The small domain is composed of two loops extending from the large domain (connecting β3 and α6) and three β pleated sheets (β4, β5, and β6). The small ion binding domain contains a zinc-binding motif: the sequence of Cys-X-X-Cys-X15-20-Cys-X-X-Cys with a 10-residue insert between the second set of cysteines, resulting in a highly flexible extended loop [18]. The SIRTs’ NAD^+^ binding pocket has been divided into three elements, A, B, and C. Site A is responsible for the binding of the adenosine moiety, while site B binds the nicotinamide ribose moiety. The catalytic reaction takes place in the hydrophobic gap between the two domains, and SIRT6 is the only SIRT that can bind NAD^+^ in the absence of an acylated protein substrate [19]. Unlike the rest of the SIRTs, SIRT6 lacks the single helix that connects the Rossmann domain and the zinc-binding motif and lacks a flexible NAD^+^ binding loop; instead, it has a stable single helix. Therefore, it is likely that SIRT6 can bind NAD^+^ without the involvement of an acetylated substrate, unlike other SIRTs [18]. Additionally, SIRT6 has an elongated acyl channel, allowing it to hydrolyze fatty-acyl groups with higher efficiency than the acetyl group in vitro [19]. The deacetylation reaction catalyzed by SIRT6 is presented in Figure 2.

## 3. Importance of Protein Deacetylation by SIRT6

The pleiotropic action of SIRT6 as both (NAD^+^)-dependent histone deacetylase (HDAC) and mono-ADP-ribosyltransferase allows it to directly or indirectly influence important processes related to histone modification, DNA damage repair, cell homeostasis, and apoptosis (Figure 1) [20]. Deacetylases, such as SIRT6, are proteins that take off acetyl groups from other proteins, particularly from histones in chromatin. Histones are proteins that aid in the packaging of DNA into the small, chromatin-like structure that is present in the nucleus of eukaryotic cells. SIRT6 acts as a histone deacetylase, causing the detachment of the acetyl group from the lysine residues of the H3 histones especially at sites H3K9, H3K18, H3K27, and histone H4, allowing SIRT6 to regulate metabolically important pathways [17].

It was discovered that SIRT6 is essential for preserving genomic integrity and inhibiting cellular aging. It interacts with acetylated histone H3 lysine 9 (H3K9ac) at chromosomal ends known as telomeres and propagates the specialized state of chromatin, which is required for the proper function and metabolism of telomeres [16]. Additionally, it was found that in cells with reduced expression of SIRT6, the hyperacetylation of H3K56 at telomeres disrupts the structure of telomeric chromatin, resulting in genomic instability [17]. SIRT6 also deacetylates pericentric chromatin, where histone H3 lysine 18 acetylation (H3K18ac) is present. This modification helps to prevent mitotic errors that may result in genomic instability and chromosomal abnormalities leading to accelerated cellular senescence and age-related illnesses [17].

Apoptosis, a natural process of programmed cell death that takes place in multicellular organisms to get rid of damaged or undesirable cells, is regulated by SIRT6 [21]. Apoptosis is controlled by a complex network of signaling pathways involving many proteins, including pro-apoptotic factors like Bax and anti-apoptotic ones like Bcl-2. SIRT6 is able to modulate apoptosis via its deacetylase activity. After DNA damage, SIRT6 can promote apoptosis through the mono-ADP-ribosylation of p73 and p53 in various cancer cells [22]. In hepatocellular carcinoma (HCC) cells, the overexpression of SIRT6 impairs cancer cell proliferation via the inhibition of ERK1/2 signaling and induces apoptosis by increasing cleaved caspase-3 levels [23]. On the other hand, SIRT6 deacetylates H3K9Ac at the promoter of the Bax, resulting in evasion from apoptosis in HCC [24]. Moreover, the depletion of SIRT6 may activate apoptosis through the hyperacetylation of the Bax promoter, the recruitment of p53, and the activation of Bax and its downstream effectors [25]. In glioma cancer cells, the restoration of SIRT6 leads to apoptosis via the upregulation of cleaved caspase-8 and Bax, inhibition of the JAK2/STAT3 pathway, and Bcl-2 downregulation [26]. SIRT6 is downregulated in nasopharyngeal carcinoma and its restoration promotes apoptosis through decreasing levels of NF-κB and Bcl-2, along with enhanced levels of cleaved caspase-3 and pro-apoptotic Bax [27]. Hence, SIRT6 may influence the balance between pro- and anti-apoptotic proteins and eventually govern apoptosis via the modulation of p53 and NF-κB signaling. This, in turn, has significant effects on a number of biological functions, such as tissue homeostasis, development, and aging, as well as the number of diseases, including cancer and neurodegenerative disorders [15].

SIRT6 mono-ADP-ribosyltransferase activity supports the ribosylation of chromatin lysine and arginine residues to facilitate DNA repair and stabilize heterochromatic regions [15]. Ribosylation of poly (ADP-ribose) polymerase 1 (PARP-1) on lysine 521 by SIRT6 enhances PARP-1 activity, thus promoting the repair of DNA damage [17]. Additionally, SIRT6 stimulates the localization of SHF2H and DNA-PKc to double-strand DNA breaks (DSBs) to maintain genomic stability [14]. Moreover, SIRT6 has other enzymatic properties, such as the removal of long-chain fatty acids from protein lysine residues, which enables the regulation of protein secretion, e.g., tumor necrosis factor-alpha (TNF-α) [17].

### 3.1. The Role of SIRT6-Mediated Protein Deacetylation in Carcinogenesis

#### 3.1.1. The Role of SIRT6 as an Oncogene

SIRT6 plays a dual function in the development of cancer that can be connected to its ability to control different signaling pathways. For instance, it has been shown that SIRT6 controls the activity of oncogenes, e.g., MYC, and tumor suppressors, e.g., TP53 [28].

Along with β-catenin, SIRT6 participates in a number of cellular signaling pathways, including those that contribute to cancer development [29]. Research performed on colorectal, breast, ovarian, hepatic, lung, and other malignancies discovered the correlation between SIRT6 expression, tumor growth, and clinical outcome. SIRT6 knockdown inhibited the migration and invasion of ovarian cancer (OC) cells OVCAR3 and OVCAR5, but did not suppress cell proliferation. The SIRT6-dependent invasiveness of OC cells was linked with the expression of EMT-related molecules such as activated β-catenin, snail, vimentin, E-cadherin, and N-cadherin. Moreover, SIRT6 and active β-catenin expressions were correlated with higher histologic grades, tumor stages, and platinum resistance in human ovarian carcinomas. Additionally, SIRT6 nuclear expression and activated β-catenin were discovered as independent indicators of shorter overall survival (OS) of patients with OC [29]. In the skin, SIRT6 functions as an oncogene, encouraging survival and proliferation. According to research, SIRT6 is a target of miR-34a in keratinocytes and is upregulated in human SCC [30]. It was discovered that the expression of SIRT6 increases in skin keratinocytes after exposure to UVB light via AKT pathway activation, and human squamous cell carcinomas (SCCs) have increased SIRT6 expression [31]. Skin-specific SIRT6 deletion inhibits proliferation and epidermal hyperplasia through downregulation of the pro-survival and pro-inflammatory protein COX-2. COX-2 has been identified as an oncogene in skin cancer, and COX-2 activity in epithelial cells plays an important role in skin cancer induced by UVB [32]. The expression of COX-2, an enzyme implicated in inflammation, proliferation, and survival, is stimulated in keratinocytes by SIRT6. These findings indicate that SIRT6 has an oncogenic function in keratinocytes and point to SIRT6 as a viable target for cancer prevention to lessen the incidence of skin cancer [31].

#### 3.1.2. The Role of SIRT6 as a Tumor Suppressor

SIRT6 promotes apoptotic cell death when DNA damage is present, preventing damaged cells from proliferation [33]. The downregulation of SIRT6 has been shown to promote tumor development and invasiveness in vivo. Importantly, loss of SIRT6 expression may lead to tumor formation even without the activation of known oncogenes and transformed SIRT6-deficient cells exhibit increased cancer growth and glycolysis, suggesting that SIRT6 plays an important role in both the establishment and maintenance of tumors. A metabolic shift towards glycolysis known as the Warburg effect, which is crucial for maintaining fast tumor development, was also shown to be mediated by SIRT6. The deletion of SIRT6 in vivo increases the number, growth, and aggressiveness of tumors and SIRT6 expression can predict patients’ survival, indicating that SIRT6 acts as a potent tumor suppressor that inhibits cancer metabolism [34].

Loss of SIRT6 causes the recruitment of Myc and the activation of the downstream let-7 target genes (*IGF2BP1* and *HMGA2*) and IGF2BP3, which stimulates PDAC development and metastasis [28]. It was shown that dysregulated USP10 function promotes carcinogenesis via SIRT6 degradation in human colon cancer and the crosstalk between UPS10 and SIRT6 controls cell-cycle progression and proliferation. USP10, a ubiquitin-specific peptidase, decreases SIRT6 ubiquitination and protects SIRT6 from proteasomal degradation. Additionally, USP10 suppresses the transcriptional activity of c-Myc oncogenes through SIRT6 and p53, which inhibits colon cancer cell-cycle progression, growth, and tumor formation [35].

The c-Jun/c-Fos/SIRT6 pathway controls the liver cancer initiation and survival of initiated cancer cells. c-Fos stimulates SIRT6 transcription, which downregulates survivin by lowering histone H3K9 acetylation and the activation of NF-κB. Increasing the SIRT6 expression or targeting the anti-apoptotic function of survivin at the cancer initiation stage noticeably impairs tumor development [36]. It was shown that SIRT6 functions as a tumor suppressor in hepatocellular carcinoma (HCC) through the binding and deacetylating of nuclear pyruvate kinase M2 (PKM2), which suppresses the oncogenic functions of PKM2, resulting in decreased cancer cell proliferation, migration, and invasiveness [37]. SIRT6 is downregulated in human liver cancer, and SIRT6 knockout in HCC induces oncogenic changes including global hypomethylation, decreased apoptosis, and metabolic changes, such as increased fat deposition and hypoglycemia [38]. SIRT6 is also downregulated in OC tissues, and SIRT6 overexpression decreases the proliferation of SKOV3 and OVCAR3 OC cells through the suppression of Notch 3 [39].

In breast cancer (BC), the progression and aggressiveness of the disease are significantly influenced by metabolic reprogramming, a hallmark of cancer characterized by the activation of genes promoting aerobic glycolysis and the suppression of mitochondrial oxidative phosphorylation. The transcription factor RUNX2, known for mediating BC metastasis to bone, plays a pivotal role in this metabolic shift and is regulated by glucose availability. This study reveals that RUNX2 expression in luminal BC cells is associated with several oncogenic features: lower levels of estrogen receptor-α (ERα), anchorage-independent growth, increased expression of glycolytic genes (such as LDHA, HK2, and GLUT1), heightened glucose uptake, and sensitivity to glucose starvation. However, these cells do not show sensitivity to inhibitors of oxidative phosphorylation. This suggests that RUNX2 expression fosters a glycolytic phenotype, typical of many cancer cells, which is known as the Warburg effect. Conversely, the knockdown of RUNX2 in triple-negative BC cells leads to a decrease in these glycolytic markers and an increase in pyruvate dehydrogenase-A1 (PDHA1) mRNA and enzymatic activity, indicating a reduction in glycolytic potential. This finding underscores the role of RUNX2 in promoting glycolytic metabolism in BC cells. SIRT6, an NAD-dependent histone deacetylase and a recognized tumor suppressor, emerges as a critical regulator of these RUNX2-mediated metabolic changes. The study shows that RUNX2 expression elevates the levels of glycolytic proteins such as pAkt, HK2, and PDHK1, which are reduced by the ectopic expression of SIRT6. This indicates that SIRT6 acts to counterbalance the effects of RUNX2, promoting a shift away from glycolysis. Furthermore, RUNX2 represses mitochondrial oxygen consumption rates (OCRs), a measure of oxidative phosphorylation. The overexpression of SIRT6, however, increases respiration in RUNX2-positive cells, while the knockdown of SIRT6 in cells with low RUNX2 expression decreases respiration. This suggests that SIRT6 plays a role in maintaining mitochondrial function and oxidative phosphorylation, which are typically suppressed in cancer cells. Importantly, the study finds that RUNX2 represses SIRT6 expression at both the transcriptional and post-translational levels. In malignant BC tissues or cell lines with high levels of RUNX2, endogenous SIRT6 expression is lower, further supporting the role of RUNX2 in suppressing SIRT6 and promoting glycolytic metabolism conducive to cancer progression [40].

Taken together, it was discovered that SIRT6 may act as a tumor suppressor by regulating the expression of several genes involved in DNA repair, cell cycle progression, and apoptosis, all of which are essential for preserving genomic integrity and preventing the growth of cancer. The differential expression of SIRT6 in various cancer types was presented in Table 1.

## 4. Screening of SIRT6 Inhibitors and Activators

SIRT6 is overexpressed in various types of cancer and its inhibition may have anti-cancer effects. On the other hand, in SIRT6-deficient cancers, anti-tumor effects may be induced by the activation of SIRT6. Therefore, the ability to regulate SIRT6 activity through inhibitors or activators makes SIRT6 a promising therapeutic target. The most important regulator of SIRT6 activity is cellular NAD^+^, which is the rate-limiting reagent [41]. It has been shown that inhibitors associate with SIRT6 close to its NAD^+^ nicotinamide binding site, which is a highly conserved site between the large Rossman domain and the minor zinc-binding domain. SIRT6 activators attach to the activator binding site that is located next to the acylated peptide substrate binding site, and they can induce conformational changes in the loop that participates in forming the acetyl-lysine binding tunnel to improve the binding of acylated substrates [42].

Studies performed on numerous cell lines showed that catechins and epicatechins are strong inhibitors of SIRT6, while anthocyanidins act as activators. Interestingly, the influence of flavonoids on SIRT6 activity depends on their concentration [42].

### 4.1. SIRT6 Activators

#### 4.1.1. 4H-Chromen

4H-chromen, a synthetic compound with a similar chemical structure to quercetin, was able to increase SIRT6 deacetylase activity over 30-fold. According to a kinetic analysis, 4H-chromen enhances SIRT6 deacetylation in an analogous mechanism to small molecule activators. It binds to the site in the hydrophobic pocket containing the catalytic center of the deacetylation reaction. It was suggested that 4H-chromene increases the affinity of the substrates to the active site, thereby stimulating the SIRT6-mediated deacetylation process. Moreover, 4H-chromen inhibits the proliferation of breast cancer (BC) cells by arresting the cell cycle in the G1 phase in MDA-MB-468, ZR-75-1, and HCC-1937 TNBC cells, but in the S/G2 phase in Hs578T cells. The reasons for these observations are not fully explained, but these findings may indicate the opposite roles of SIRT6 at different stages of BC carcinogenesis and in different types of breast cancer [43].

#### 4.1.2. MDL-811

MDL-811, a newly discovered allosteric activator of SIRT6, promotes EZH2 deacetylation and FOXC1 expression, resulting in the amelioration of brain ischemic injury, neuroinflammation, and improved stroke outcomes [44]. According to a docking analysis, MDL-811 binds to the SIRT6 allosteric pocket through hydrogen bonds with Phe86 and participates in hydrophobic interactions with Val153 and Phe86 [44]. In colorectal cancer (CRC) cells, MDL-811 enhances SIRT6-mediated histone H3 deacetylation (H3K9Ac, H3K18Ac, and H3K56Ac) and inhibits the proliferation of CRC cell lines and organoids derived from patients [45]. Additionally, gene set enrichment analysis (GSEA) revealed that MDL-811 changes the expression of genes responsible for the inhibition of DNA replication and cell cycles. The treatment of CRC cells with MDL-811 reduced the expression of genes involved in glucose metabolism mediated by Hif1α (LDHA, PDK1, PKM2, and GLUT1), cell cycle control (PCNA, CCNA2, CDC2, and CDC25C), and genes responsible for IGF signaling (MTOR, AKT1, and AKT2). In vitro studies showed that this compound induced inhibition of the cell cycle in the G0/G1 phase, which confirms that the activation of SIRT6 inhibits cell cycle progression in cancer cells [45]. The antitumor activity of MDL-811 has been also demonstrated in CRC patient-derived organoids, patient-derived and cell-line-derived xenograft models, as well as in a spontaneous mouse model of CRC [46].

#### 4.1.3. MDL-800

MDL-800 directly binds to the SIRT6 surface allosteric site and enhances its deacetylation activity [47]. MDL-800 activates SIRT6 by enhancing the binding affinity of cofactors and acylated substrates as well as by increasing the SIRT6 catalytic efficiency. In a mouse model, SIRT6 alleviated liver fibrosis by the deacetylation of Smad2, inhibited the TGF-β-induced phosphorylation and nuclear localization of Smad2, and decreased the transcription of TGF-β/Smad2 signaling [48]. In human HCC cells, the interaction of MDL800 with SIRT6 enhances SIRT6 deacetylase activity up to 22-fold, leading to a global decrease in the acetylation of H3K9 and H3K56. Consequently, MDL-800 inhibits HCC cells’ proliferation through cell-cycle arrest driven by SIRT6 and shows antitumor activity in mice tumor xenograft models. These findings demonstrate that the activation of SIRT6 by small molecule activators is a potential therapeutic approach for HCC treatment [47].

#### 4.1.4. Fluvastatin

Fluvastatin is a potent inhibitor of 3-hydroxy-3-methylglutaryl coenzyme A (HMG-CoA) reductase, which is used for the treatment of cardiovascular diseases and hypercholesterolemia. In addition, it shows promising activity against breast, liver, and endometrial cancer cells. Fluvastatin stimulates SIRT6-dependent deacetylation of histone H3 (H3K9ac and H3K56ac) in HepG2 cells. X-ray crystallography revealed that this compound acts by attaching to the SIRT6 substrate acyl exit site and then forming an SIRT6/ADP-ribose/fluvastatin complex [19]. Fluvastatin inhibits proliferation, migration, and invasion, and induces apoptosis in endometrial cancer (EC) cells by upregulating SIRT6 expression. The upregulation of SIRT6 by fluvastatin increases p53 and cleaves caspase 3 levels [49].

### 4.2. SIRT6 Inhibitors

#### 4.2.1. Quercetin

Quercetin, a flavonoid found in various types of vegetables and fruits, exhibits different pharmacological effects, including antitumor activity [50]. Quercetin can modulate SIRT6 activity, acting as an inhibitor at low concentrations and an activator at high concentrations. Crystal structure analyses and binding assays showed that quercetin-based compounds activate SIRT6-mediated deacetylation via binding to the SIRT6-specific acyl binding channel. In addition, quercetin has been shown to inhibit SIRT6-mediated demyristoylation activity by binding to the distal site of the SIRT6 acyl channel [51]. On the other hand, two quercetin derivatives, 2-chloro-1,4-naphtoquinone-quercetin and diquercetin, were identified as SIRT6 inhibitors. Molecular docking studies revealed that diquercetin binds to the SIRT6 nicotinamide moiety binding site, while 2-chloro-1,4-naphtoquinone-quercetin docks into the substrate binding site. The results of molecular modeling and in vitro studies suggest that diquercetin competes with NAD^+^, while 2-chloro-1,4-naphthoquinone-quercetin competes with the acetylated substrate in the SIRT6 catalytic site [33]. In liver hepatoblastoma (HB) cell lines, quercetin has been shown to increase SIRT6 expression, leading to the deacetylation of histone H3K9, the downregulation of Frizzled Class Receptor 4 (FZD4), and the inhibition of the activation of the Wnt/β-catenin pathway. Quercetin inhibited the proliferation and invasion of HB cells through the Wnt/β-catenin pathway and promoted apoptosis. Additionally, quercetin suppressed tumor growth in a mouse xenograft model [50].

#### 4.2.2. Quinazolinedione Derivatives

Quinazolinedione derivatives were found to inhibit SIRT6 activity in the micromolar range but had moderate SIRT6/SIRT1 selectivity. These compounds exhibited a competitive inhibition pattern against the NAD^+^ cofactor by binding to the active site of SIRT6 and were able to increase histone H3K9 acetylation. Since SIRT6 cooperates with Poly [ADP-ribose] polymerase 1 (PARP-1) in the repair of DNA double-strand breaks, the effect of their inhibition was assessed in Capan 1 pancreatic cancer (PaC) cell lines with BRCA-2 deficiency. The inhibition of SIRT6 activity with quinazolinedione derivatives potentiated the anti-cancer effect of olaparib, a PARP-1 inhibitor, by inhibiting the growth of Capan-1 cells. Additionally, treatment of PaC cell line BxPC-3 with both inhibitors decreased the synthesis of tumor necrosis factor α (TNF-α) and increased glucose uptake [52].

## 5. Summary of Sirtuins as a Potential Therapeutic Target

Sirtuins (SIRTs), a family of NAD^+^-dependent deacetylases, play a pivotal role in regulating cellular processes, including the pathogenesis of cancers. Their involvement in these cancers is complex and multifaceted, influencing aspects like chemoresistance, metastasis, and overall disease progression [53]. In the context of HNSCC, SIRT1, SIRT3, and SIRT7 have been identified as significant factors, yet their precise mechanisms of action remain partially understood. SIRT1, in particular, demonstrates a dual role in HNSCC, with lowered expression in cancer cells and a potential contribution to chemoresistance. Understanding how SIRT1 influences the response of cancer cells to chemotherapy, especially drugs like cisplatin, is crucial. The modulation of SIRT1 activity could significantly impact the efficacy of cancer treatments. The overexpression of SIRT3 in oral cancers suggests its potential as a therapeutic target. Research into SIRT3 inhibitors could provide new insights into enhancing the effectiveness of existing HNSCC treatments. Additionally, the impact of these inhibitors on normal human oral keratinocytes, particularly in inducing apoptosis and mitochondrial dysfunction, warrants investigation [54]. The roles of SIRTs 2, 4, 5, and 6 in HNSCC are less explored. Future studies should focus on determining their specific roles in the pathogenesis of HNSCC and their potential as therapeutic targets. Understanding the expression patterns of SIRTs in different HNSCC subtypes, such as cancers of the lip, tongue, and other oral areas, is crucial for comprehending the heterogeneity of the disease and developing targeted therapeutic strategies. SIRTs also serve as potential epigenetic targets in HNSCC therapy. Developing and testing epigenetic modulators of SIRTs could lead to new therapeutic strategies that enhance chemotherapy efficacy and limit cancer cell proliferation. Investigating the interactions of SIRTs with other molecular factors, such as miRNA, p53, TGF-β, and NF-kβ, is essential for a complete understanding of their complex roles in cancer progression and treatment response [55].

In breast cancer, the mechanisms of SIRT1 in chemoresistance, particularly its role in amplifying MDR1 gene expression and mediating drug resistance, need further exploration [56]. The controversial status of SIRT3 in BC, its association with tamoxifen resistance, and its impact on ROS generation and antioxidant activity, also require in-depth study. The potential of SIRT6 as a therapeutic target in BC, especially given its association with poorer overall survival, is another area of interest. The development of novel SIRT6 inhibitors could enhance the efficacy of chemotherapeutics like gemcitabine [57].

The potential of sirtuin modulators, such as amurensin G and Psammaplin A, in reversing MDR and enhancing the efficacy of chemotherapeutics in BC, presents a promising avenue for treatment. Combining these modulators with traditional chemotherapy could offer a novel approach to overcome MDR and metastasis in BC. Addressing the heterogeneity of BC through subtype-specific therapeutic strategies based on differential SIRT expression is crucial. The concept of polypharmacology in sirtuin targeting, aiming to find the right balance in specificity, potency, and toxicity for therapeutic use in BC, is another promising research direction. Utilizing SIRTs as predictive models for BC management could help in predicting survival in patients with metastatic BC and those suffering from MDR. In conclusion, the complex role of SIRTs in cancers like HNSCC and BC, particularly in the context of drug resistance and metastasis, presents both challenges and opportunities. Future research should aim to unravel these complexities, developing targeted therapies that leverage the unique properties of SIRTs to improve cancer management and patient outcomes [58].

## 6. Conclusions and Future Perspectives

Sirtuins (SIRTs), class III histone deacetylases (HDACs), are a group of highly conserved proteins that play an important role in the regulation of cell proliferation, migration, metastasis, and apoptosis. One of the lesser-known sirtuins is SIRT6, which has both (NAD^+^)-dependent deacetylase and mono-ADP-ribosyl transferase activities. This protein is involved in the control of different pathways responsible for cancer development. The role of SIRT6 in carcinogenesis is complex as this deacetylase is differentially expressed in different types of cancer cells compared to normal cells. Dynamic changes in the level of SIRT6 in neoplastic cells indicate its important role in the process of carcinogenesis and cancer progression. The regulation of SIRT6 activity by treatment with small molecule modulators can inhibit proliferation and invasion, induce apoptosis, sensitize cancer cells to existing anti-cancer agents, and decrease tumor growth in vivo. Therefore, further research is needed to better understand the role of SIRT6 in cancer initiation and progression. Moreover, the discovery of novel SIRT6 inhibitors and activators may help to develop more effective anti-cancer therapeutic treatments.

Future research on SIRT6 holds significant promise for cancer therapy. One key area of focus will be elucidating the molecular mechanisms by which SIRT6 influences cancer cell biology. This includes understanding how SIRT6 interacts with other cellular proteins and signaling pathways to regulate cancer cell behavior. Advanced techniques such as proteomics and transcriptomics could provide deeper insights into these interactions and the downstream effects of SIRT6 modulation.

Another important aspect will be the development of more specific and potent SIRT6 modulators. Current inhibitors and activators of SIRT6 offer a starting point, but there is a need for compounds with higher specificity and efficacy. The use of high-throughput screening methods and structure-based drug design could accelerate the discovery of such molecules. Additionally, the role of SIRT6 in the tumor microenvironment and its interaction with immune cells is an area that warrants further exploration. Understanding how SIRT6 affects the immune response to cancer could open new avenues for combination therapies where SIRT6 modulators are used alongside immunotherapies. Clinical trials are also crucial for translating these findings into therapeutic applications. The safety, efficacy, and optimal dosing of SIRT6 modulators must be established in a clinical setting. Moreover, identifying biomarkers for SIRT6 activity could help select the patients that are most likely to benefit from SIRT6-targeted therapies.

## Figures and Tables

**Table 1:** Differential expression of SIRT6 in various cancer types.

Type of Cancer	Level of SIRT6 Expression
Ovarian Cancer (OC)	↑
Skin Cancer (SCC)	↑
Hepatocellular Carcinoma (HCC)	↓
Breast Cancer (BC)	↓
